# Identification of the sAPRIL Binding Peptide and Its Growth Inhibition Effects in the Colorectal Cancer Cells

**DOI:** 10.1371/journal.pone.0120564

**Published:** 2015-03-31

**Authors:** Xiao-qing He, Jing Guan, Fang Liu, Jing Li, Mei-rong He

**Affiliations:** 1 Guangdong Provincial Key Laboratory of Gastroenterology, Institute of Digestive Diseases, Nanfang Hospital, Southern Medical University, Guangzhou 510515, Guangdong Province, China; 2 Oncology Department, Wuzhou Red Cross Hospital, Wuzhou 543002, Guangxi Province, China; 3 Army Reserve Anti-aircraft Artillery Hospital, Zhengzhou 450002, Henan Province, China; National Health Research Institutes, TAIWAN

## Abstract

**Background:**

A proliferation-inducing ligand (APRIL) is a member of the tumor necrosis factor (TNF) super family. It binds to its specific receptors and is involved in multiple processes during tumorigenesis and tumor cells proliferation. High levels of APRIL expression are closely correlated to the growth, metastasis, and 5-FU drug resistance of colorectal cancer. The aim of this study was to identify a specific APRIL binding peptide (BP) able to block APRIL activity that could be used as a potential treatment for colorectal cancer.

**Methods:**

A phage display library was used to identify peptides that bound selectively to soluble recombinant human APRIL (sAPRIL). The peptides with the highest binding affinity for sAPRIL were identified using ELISA. The effects of sAPRIL-BP on cell proliferation and cell cycle/apoptosis *in vitro* were evaluated using the CCK-8 assay and flow cytometry, respectively. An *in vivo* mouse model of colorectal cancer was used to determine the anti-tumor efficacy of the sAPRIL-BP.

**Results:**

Three candidate peptides were characterized from eight phage clones with high binding affinity for sAPRIL. The peptide with the highest affinity was selected for further characterization. The identified sAPRIL-BP suppressed tumor cell proliferation and cell cycle progression in LOVO cells in a dose-dependent manner. *In vivo* in a mouse colorectal challenge model, the sAPRIL-BP reduced the growth of tumor xenografts in nude mice by inhibiting proliferation and inducing apoptosis intratumorally. Moreover, in an *in vivo* metastasis model, sAPRIL-BP reduced liver metastasis of colorectal cancer cells.

**Conclusions:**

sAPRIL-BP significantly suppressed tumor growth *in vitro* and *in vivo* and might be a candidate for treating colorectal cancers that express high levels of APRIL.

## Introduction

Colorectal cancer is one of the most common digestive cancers worldwide and patients often die from cancer cell metastasis [1]. Traditional chemotherapy has disadvantages including, different degrees of cell cytotoxicity and off target effects that can damage healthy tissues, these side effects have a detrimental impact on patient quality of life. It is crucial to develop a targeted cancer therapy that has low cytotoxicity and is highly selective to improve the prognosis and survival rate of colorectal cancer patients.

A Proliferation-Inducing Ligand (APRIL) is a ligand in the tumor necrosis factor (TNF) superfamily that functions as a soluble factor [2]. APRIL is primarily expressed by hematopoietic cells and has biological roles in B cell survival and T cell activation [3–5]. Normal tissues express very low level of APRIL however, cancer cell lines and tumors such as digestive cancer, hematological malignancy, and urothelial cancer, express high levels of APRIL [6–9]. APRIL is involved in multiple process related to tumorigenesis such as promoting tumor cell proliferation and survival in various types of cancer [10–14]. In colorectal cancer, high expression of APRIL is closely correlated with tumor growth, metastasis, and 5-fluorouridine (5-FU) resistance [12, 13, 15].

APRIL exerts its biological functions by interacting with several receptors. Known APRIL receptors includes B cell maturation antigen (BCMA), transmembrane activator and cyclophilin ligand interactor (TACI), and heparin sulfate proteoglycans (HSPGs) [7, 16]. BCMA is primarily expressed on mature B lymphocytes [17], but is also highly expressed on multiple myeloma cells [18]. TACI is expressed primarily on mature B cells and plasma cells [19], and HSPGs are widely expressed on the surface of many mammalian cells [20]. Upon binding to these receptors, APRIL enhances proliferation, or suppresses apoptosis to promote tumor progression through multiple molecular mechanisms. In B-cell chronic lymphocytic leukemia (B-CLL), soluble APRIL stimulates NF- μB activation, and protects B-CLL cells from spontaneous or drug-induced apoptosis [21]. In non-Hodgkin's lymphoma (NHL) B cells, recombinant APRIL activates NF- μB, upregulates the anti-apoptotic proteins Bcl-2 and Bcl-xL, and downregulates the pro-apototic protein Bax to inhibit apoptosis [14]. In glioma cells, ectopic expression of APRIL confers protection from death ligand/receptor-mediated apoptosis possibly by upregulating anti-apoptotic protein X-linked inhibitor of apoptosis protein (XIAP) [22]. In multiple myeloma, APRIL promotes cell cycle progression by increasing S phase and G_2_-M phase [23]. In human colorectal cancer cells, knocking down APRIL using RNA interference blocks transforming growth factor (TGF)-μ1 signaling and activation of extracellular signal-regulated kinases (ERK) to induce cell cycle arrest and apoptosis [24].

Targeting APRIL to suppress tumor growth, proliferation, and survival could be a feasible strategy to treat colorectal cancer. Others have shown that silencing APRIL reduces tumor cell proliferation and metastasis in colorectal cancer [25–27]. We have previously shown that downregulation of APRIL reduces proliferation, increases apoptosis, and enhances sensitivity to 5-FU chemotherapy in the colorectal cell line LOVO, which expresses high levels of APRIL [28]. Gao *et al*. have successfully developed an anti-APRIL monoclonal antibody and demonstrated its anti-proliferative effect *in vitro* and *in vivo* [29]. Other groups have used recombinant sAPRIL receptors or mutant sAPRIL to compete with endogenous sAPRIL for binding to its receptors [30, 31]. Compared to other strategies for targeting APRIL, such as siRNA or anti-APRIL antibodies, polypeptides possess several advantages including low immunogenicity, a high degree of safety, ease of synthesis and purification, the ability to penetrate to tissue and organs, and less toxicity. Identifying and synthesizing anti-tumor polypeptides has become a popular strategy for developing targeted anti-cancer therapies [32, 33]. However, no APRIL specific polypeptides have been reported to date. Therefore, the aim of this study was to identify sAPRIL specific binding peptides using a phage display library, and to evaluate its *in vitro* and *in vivo* anti-cancer effects to provide a therapeutic candidate for use against colorectal cancers that express high levels of APRIL.

## Materials and Methods

### In vitro panning

Human recombinant sAPRIL (R&D, USA) was used as the target protein for screening from a phage display 12-peptide library according to manufacturer’s protocol (NEB, USA). Briefly, phages from the library were added onto sAPRIL-coated ELISA plates and incubated at room temperature for 60 min. Unbound phages were removed by washing with TBST (TBS + 0.1% [v/v] Tween-20). Subsequently, bound phages were washed off with glycine-HCl (pH 2.0) and amplified. The phages with binding affinity for sAPRIL were harvested after four rounds of binding/amplification. Except the first round, the wash buffer for removing the unbound phages was TBST (TBS + 0.5% [v/v] Tween-20).

### Selection of positive clone by ELISA

Twenty single colonies were picked from the last round of panning and incubated with the *E*. *coli* host strain ER2738 for 4.5 h at 37°C with shaking. A sAPRIL-coated ELISA plate was blocked with 5% bovine serum albumin (BSA) overnight and then supernatant from a single colony phage suspension was added for 1 h. To detect bound phages, a horseradish peroxidase (HRP)-labeled anti-M13 antibody (GE Healthcare, USA) was added for 1 h, followed by tetramethylbenzidine (TMB) for 10 min, and then the reaction was stopped with an H_2_SO_4_ solution. Colonies with an A_450_ that was at least 6 fold higher than the positive control were considered positive for sAPRIL binding.

### Sequence analysis of selected positive clones and peptide synthesis

The sequences of the positive clones were analyzed and translated into the amino acid sequences, and the corresponding peptides were synthesized and purified (Hangzhou Chinese Peptide Company). The sAPRIL binding peptides were then tested for their affinity for sAPRIL and the TNF superfamily member BAFF by ELISA. The peptide that had the highest binding activity for sAPRIL was chosen for follow-up experiments.

### Cell culture

The Colorectal colon cancer cell lines SW620, LOVO, HCT116, HT29, and SW480 were purchased from ATCC. All cell lines were cultured in RPMI-1640 with 10% FBS (Gibco BRL Co. LTd., USA), and 100 KU/L penicillin & streptomycin at 37°C with 5% CO_2_.

### In vivo model

All animal studies were conducted in accordance with institutional guidelines, and approved by the Animal Care and Use Committee at Nanfang hospital. Female BALB/c nude mice (4–6 weeks of age) were purchased from Guangdong Medical Laboratory Animal Center and maintained under gnotobiotic conditions. LOVO cells (2×10^6^ cells in 100 μL PBS) were subcutaneously injected (100 μL/injection; one injection site /tumor) and the tumor size was measured every 3 days. After 3 weeks, the subcutaneous nodule was considered a tumor when it reached 100–200 mm^3^. The mice were divided into three groups and injected intratumorally with PBS (control), 20 mg/kg sAPRIL-BP (low dose), or 40 mg/kg sAPRIL-BP (high dose) every two days for two weeks. Tumor volume (V) was calculated using the formula: V = AB^2^/2, in which A is the maximum diameter, and B is the diameter perpendicular to the line of A.

For the colorectal cancer liver metastasis model, LOVO cells (2x10^6^ cells/100 μL) were injected into the spleen after laparotomy. Three weeks after injection, mice were randomized to 3 groups (N = 5) and intraperitoneally injected with PBS (200 μL) as a control, the low dose of sAPRIL-BP (20 mg/kg), or the high dose of sAPRIL-BP (40 mg/kg) on days 22, 24, 26, 30, 32, and 34 post tumor injection. Mice were sacrificed at day 35. The livers were harvested and the number and size of metastasized nodules was determined.

### Reverse transcriptase polymerase chain reaction (RT-PCR)

RNA from the colorectal cancer cell lines was extracted with Trizol (Invitrogen, USA) according to the manufacturer’s instructions. RNA from each sample was used for cDNA synthesis using RevertAID kit (Fermentas, Canada). PCR was performed using PCR PreMix (SBS Genetech, China). The primer sequences for APRIL were: forward 5′-AGAAGAAGCAGCACTCTGTC-3′ and reverse 5′-CCATGTGGAGAGAGGTTAAG-3′ (product 394 bp). GAPDH was used as the internal control. The GAPDH primers were: forward 5′-CGACCACTTTGTCAAGCTCA-3′ and reverse 5′-AGGGTCTACATGGCAACTG-3′ (product 240 bp). The PCR reaction conditions were: 95°C for 3 min, 94°C for 50 s, 58°C for 30 s, and 72°C for 1 min, for 32 cycles, followed by extension at 72°C for 7 min. The PCR products were separated using agarose gel electrophoresis and visualized with UV irradiation.

### Western Blotting

Western blotting was performed according to standard protocols. The antibodies used were against APRIL, cyclin D1, cyclin A, cyclin E, cyclin B1, CDK4, CDK6, p53, p27, and p16 (1:1000, Abcam, USA) and rabbit anti-GAPDH (1:2000, ZSGB-BIO, China).

### Cell proliferation assay

LOVO and SW620 cells were seeded in 96-well culture plates (2×10^3^ cells/well) in a 200 μL volume overnight. Five doses of recombinant sAPRIL binding peptide (5 μM, 10 μM, 20 μM, 40 μM, and 80 μM) were added for 24 h, 48 h, and 72 h. Cell proliferation was determined using the CCK-8 kit (Beyotime Institute of Biotechnology, China) according to the manufacturer’s instructions.

### Cell cycle and apoptosis analysis

LOVO cells were seeded in 6-well culture plates (1×10^5^ cells/well/mL). After 24 h, sAPRIL binding peptide (20 μM or 40 μM) was added for an additional 48 h. For cell cycle analysis by flow cytometry (BD LSR flowcytometer, BD Biosciences, USA), the cells were harvested and fixed with 7% ethanol for 24 h at 4°C. After washing with PBS, the cells were stained with propidium iodide (PI) solution (50 μg/mL PI, 100 μg/mL RNAse A, 0.2% Triton X-100) for 30 min at 4°C. The data were analyzed using the Modfit LT software. For apoptosis analysis by flow cytometry, the harvested cells were stained with an apoptosis detection kit (Beyotime, China) according to the manufacturer’s instructions. Briefly, the cells were stained with 500 μL of binding buffer, 5 μL of Annexin V-FITC, and 5 μL of PI for 5–15 min at room temperature in the dark.

### Immunohistochemistry staining

All xenograft tumors were fixed with 10% formalin and paraffin-embedded for sectioning. For antigen retrieval, tissue sections were placed in a 0.01 M citrate buffer at pH 6.0 and then heated at 98°C to 100 for 15 min in a microwave oven. Endogenous peroxidase activity was blocked by incubating the sections in 3% hydrogen peroxide (in fresh methanol) for 15 min at room temperature. Then tissue sections were stained with primary antibodies specific for Ki-67 (1:100, Abcam, USA) and cleaved caspase-3 (1:200, CST, USA). A conjugated rabbit anti-mouse IgG secondary antibody (1:200, Abcam, USA) was used. Positive staining was visualized with DAB. Images were captured using an Olympus BX41 light microscope. The percentage of Ki67-positive tumor cells (proliferative index) and the percentage of cleaved caspase-3-positive tumor cells (apoptosis index) were determined for three separate fields containing at least 1000 adjacent cells for each slide.

### Statistics

All results were analyzed with SPSS version 17.0 software. All of the experiments were repeated at least three times. The results were expressed as mean ± standard deviation (SD) unless otherwise specified. Statistical significance was evaluated using a one-way ANOVA or two-tailed t test. The difference between groups was considered significant when *p* <0.05.

## Results

### Identification of specific sAPRIL-binding peptide

**Fig 1:**
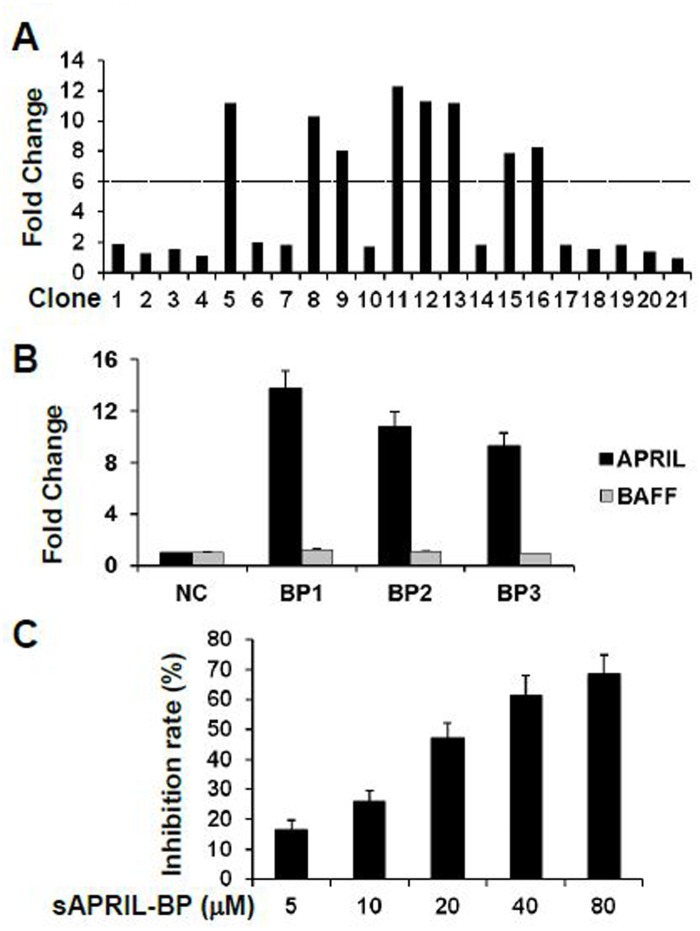
Selection of sAPRIL-BP. (A) The binding affinity of phage clones No.1–20 for sAPRIL were determined by ELISA. Clone 21 was used as a positive control. The fold change of the optical density was normalized to the positive control. Clones that had at least a 6-fold greater affinity than the positive control were considered ‘positive’ for sAPRIL binding. (B) Three binding peptides were synthesized and their binding affinity with sAPRIL (black bars) was determined and compared with the negative control (NC) using ELISA. Cross-reactivity was assessed by measuring the binding affinity to BAFF (grey bars). (C) Clone BP1 (sAPRIL-BP) was mixed with sAPRIL at different doses to compete for binding with fixed LOVO cells.

A phage library was used to identify 20 single clones expressing potential sAPRIL binding peptides. The clones were randomly selected after four rounds of panning. Eight single clones were identified as ‘positive’ indicating a high binding affinity (defined as an OD ≥6-fold the OD of the positive control) for sAPRIL by ELISA (Fig 1A). The eight clones represented three DNA sequences that correspond to the following binding polypeptides (BP): AAAPLAQPHMWA, SSTTTSDKYLSA, and SNLHDNNTEKNV (Table 1). The affinity for sAPRIL was measured for each polypeptide by ELISA and compared to an unrelated polypeptide (HWDPFSLSAYFP) as a negative control. The three peptides identified using the phage library had sAPRIL binding affinities that were 13.7, 10.8, and 9.3-fold higher than the control peptide, respectively (Fig 1B). The binding affinity of the sAPRIL-BPs was also tested against another TNF superfamily ligand BAFF (B-cell activation factor of the TNF family). The binding affinities for each sAPRIL-BP were 1.2, 1.1, and 0.9, respectively (Fig 1B). Taken together, these results indicated that the sAPRIL-BPs specifically binds to APRIL and do not cross-react with BAFF. sAPRIL-BP1 (AAAPLAQPHMWA) had the highest binding affinity and was subsequently used *in vitro* to assess whether it was able to inhibit sAPRIL binding in the fixed human colorectal cancer cell line LOVO cells (Fig 1C). sAPRIL-BP1 showed a dose-dependent inhibitory effect on sAPRIL binding to the LOVO cells. Therefore, sAPRIL-BP1 (hereafter sAPRIL-BP) was chosen for further functional characterization.

**Table 1:** DNA and amino acid sequence of positive clones.

Clone	DNA and amino acid sequence											
5, 11, 12, 13	GCT	GCG	GCT	CCG	CTT	GCG	CAG	CCT	CAT	ATG	TGG	GCG
	A	A	A	P	L	A	Q	P	H	M	W	A
8, 16	TCT	AGT	ACT	ACT	ACT	AGT	GAT	AAG	TAT	CTT	AGT	GCG
	S	S	T	T	T	S	D	K	Y	L	S	A
9	TCT	AAT	CTG	CAT	GAT	AAT	AAT	ACG	GAG	AAG	AAT	GTG
	S	N	L	H	D	N	N	T	E	K	N	V

Eight positive clones (defined as an OD ≥6-fold the OD of the positive control) out of twenty single clones expressing potential sAPRIL binding peptides were selected and the DNA sequence was analyzed. Clone 15 was eliminated due to incorrect sequence. Three DNA sequences were presented from the 7 clones and their corresponding polypeptide sequence were showed.

### Anti-proliferative effects of sAPRIL binding peptides in LOVO cells

**Fig 2:**
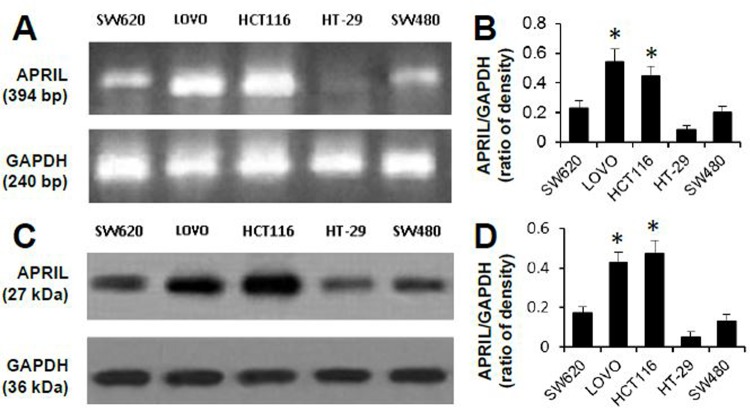
APRIL mRNA and protein expression in human colorectal cell lines. APRIL expression was assessed in five human colorectal cell lines (indicated) using RT-PCR (A) and Western Blotting (C). Representative gel images are shown. GAPDH was used as the internal control. The optical densities of the APRIL mRNA (B) and protein (D) bands were analyzed and normalized to the internal control. *P<0.05 compared to SW620, HT-29, or SW480.

**Fig 3:**
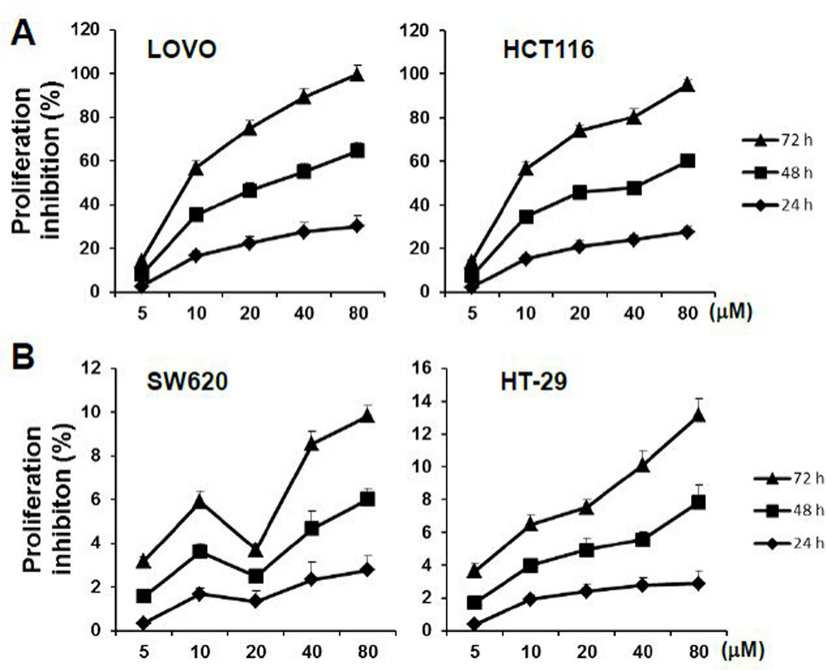
Effect of sAPRIL-BP on the proliferation of LOVO and SW620 cells. (A) APRIL^high^ LOVO and HCT116 cells and (B) APRIL^low^ SW620 and HT-29 cells were treated with the indicated doses of sAPRIL binding peptides for 24, 48, and 72 h, and proliferation was determined using the CCK-8 kit. The rate of proliferation inhibition was calculated as: (%) = [(mean of OD_control_—mean of OD_experimental_) / mean of OD_control_]×100%.

To evaluate the role of APRIL in colorectal cancer, APRIL expression was examined in five human colorectal cell lines by RT-PCR (Fig 2A and 2B) and western blotting (Fig 2C and 2D). The HCT116 and LOVO cell lines had significantly higher levels of APRIL mRNA (*p*<0.05) and protein (*p*<0.05) than the SW480, SW620, and HT29 cell lines. Therefore, the effect of sAPRIL-BP was evaluated in LOVO and HCT116 (APRIL^high^) cells, and SW620 and HT-29 (APRIL^low^) cells. To determine whether there were dose effects, cell proliferation was measured at different concentrations of sAPRIL-BP. The rate of proliferation was significantly inhibited (*p<*0.05) by treatment with sAPRIL-BP in LOVO and HCT116 cells (Fig 3A) in a time- and dose-dependent manner. However, this was not the case in SW620 and HT-29 cells (Fig 3B). There results suggest that the anti-proliferative effects of sAPRIL-BP are specific for APRIL^high^ cells.

### Effects of sAPRIL binding peptides on the cell cycle and apoptosis in LOVO cells

**Fig 4:**
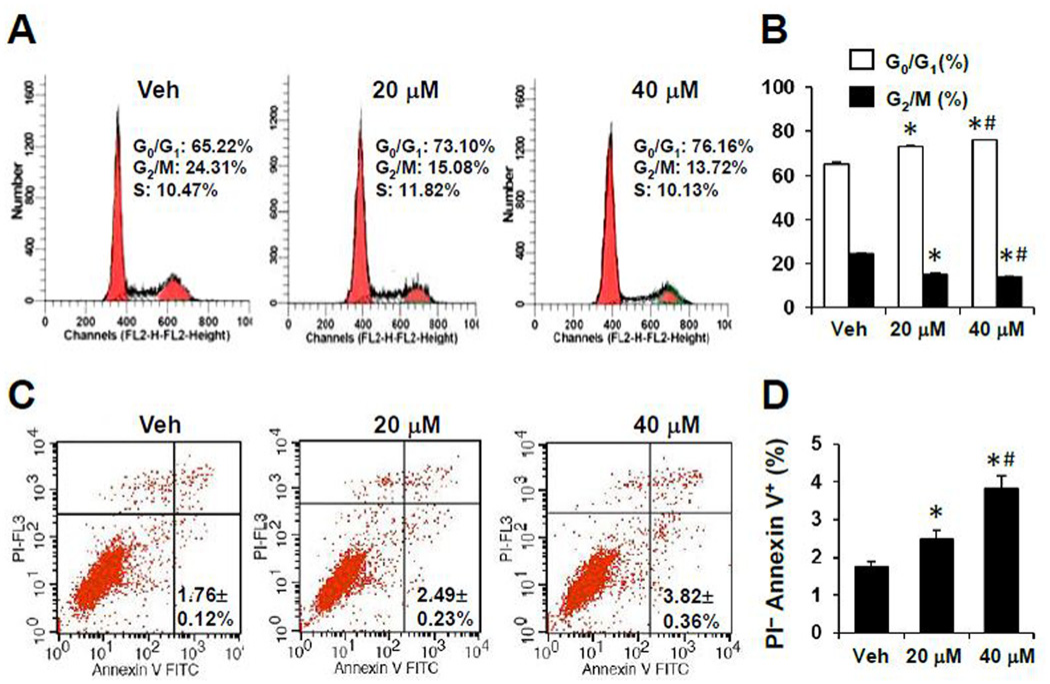
Effect of sAPRIL-BP on cell cycle and apoptosis of LOVO cells. LOVO cells were treated with the indicated doses of sAPRIL-BP for 48 h. Cells were stained with PI for cell cycle analysis (A and B) and PI + Annexin V for apoptosis analysis (C and D) by flow cytometry. *p <0.05 compared to Vehicle control; #*p* <0.05 compared to the low dose group.

**Fig 5:**
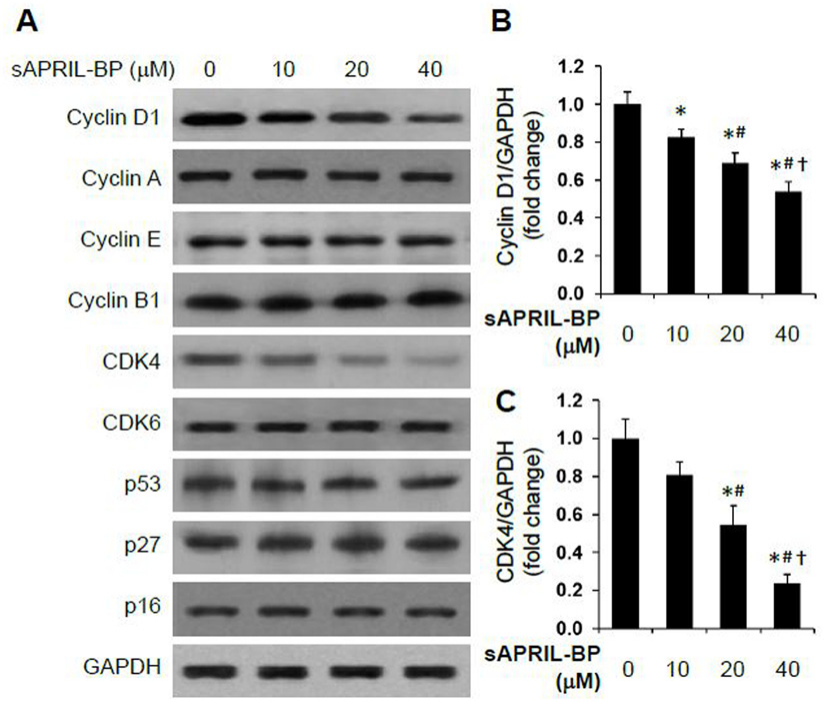
Effect of sAPRIL-BP on the expression of cell cycle-related proteins. LOVO cells were treated with the indicated doses of sAPRIL-BP for 48 h. (A) The expression levels of the indicated cell cycle proteins were assessed by Western Blotting analysis. GAPDH was used as the internal control. The protein size of Cyclin D1 is 34 kDa, Cyclin A 49 kDa, Cyclin E 50 kDa, Cyclin B1 55 kDa, CDK4 34 kDa, CDK6 37 kDa, p53 53 kDa, p27 27 kDa, p16 40 kDa, and GAPDH 36 kDa. The optical densities of the cyclin D1 (B) and CDK4 (C) protein bands were analyzed and normalized to the internal control as fold change. *P<0.05 compared to Vehicle group; #*P*<0.05 compared to 10 μM group, †*P*<0.05 compared to 20 μM group.

Since the proliferation of cancer cells is primarily regulated by the cell cycle, we next determined which phase of the cell cycle was affected by sAPRIL treatment. Based on the proliferation results (Fig 3A), we chose to test 20 μM and 40 μM of sAPRIL-BP in LOVO cells to determine how inhibiting sAPRIL altered the cell cycle and apoptosis. By flow cytometry, sAPRIL-BP significantly increased the percentage of cells in the G_0_/G_1_ phase at the low dose (20 μM sAPRIL-BP: 73.1 ± 0.6%; *p*<0.001) and the high dose (40 μM sAPRIL-BP: 76.2 ± 0.1%; *p*<0.001) when compared to the Vehicle control (65.2 ± 0.8%). sAPRIL-BP also significantly reduced the percentage of cells in the G_2_/M stage compared to the Vehicle control (24.3 ± 0.8%) at the low dose (20 μM sAPRIL-BP: 15.1 ± 0.5%; *p*<0.05) and the high dose (40 μM sAPRIL-BP: 13.7 ± 0.5%; *p*<0.001; Fig 4A and 4B). This suggests that the anti-proliferative effects of sAPRIL-BP in LOVO cells are due to an accumulation of cells in the G_0_/G_1_ stage, which blocks cell cycle progression. Regarding the apoptotic effects of sAPRIL-BP, flow cytometry analysis showed that sAPRIL-BP had dose-dependent effects on the percentage of LOVO cells in early apoptosis (PI^-^ Annexin V^+^ cells). Compared to the Vehicle control (1.76 ± 0.12%) the low dose (20 μM sAPRIL-BP: 2.49 ± 0.23%; *p*<0.05) and high dose (40 μM sAPRIL-BP: 3.82 ± 0.36%; *p*<0.05) significantly increased early apoptotic cells (Fig 4C and 4D). We further investigated the possible molecular mechanism underlying the effect of sAPRIL-BP on cell cycle progression. Treatment with sAPRIL-BP had no effect on cyclin A, B, E1, CDK6, p53, p27, and p16 expression (Fig 5A). But the expression of the G1/S-specific protein cyclin D1 (Fig 5A and 5B) and cell division kinase cyclin-dependent kinase 4 (CDK4) (Fig 5A and 5C) were downregulated in a dose-dependent manner by treatment with sAPRIL-BP.

### Effect of sAPRIL-BP on the xenograft tumor growth

**Fig 6:**
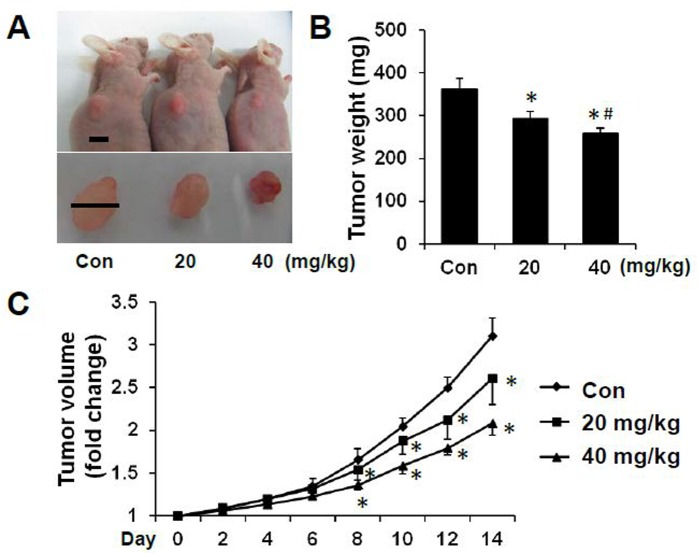
*In vivo* effects of sAPRIL-BP on tumor development. LOVO cells were injected subcutaneously into nude mice and allowed to grow for 3 weeks. Once the tumor was establishes, the mice were divided into 3 groups (N = 5) and treated with PBS (control), low (20 mg/kg), or high (40 mg/kg) dose of sAPRIL-BP every other day. (A) Representative examples of the tumors from each group are shown. Top panel: Scale bar, 8 mm. Bottom panel: Scale bar, 7 mm. (B) Mice were sacrificed after two weeks of treatment with sAPRIL-BP and the tumor weights were recorded. *p <0.05 compared to control. #*p* <0.05 compared to the low dose group. (C) The tumor volume was recorded every two days during treatment. *p <0.05 compared to control.

Then the *in vivo* effect of sAPRIL-BP was evaluated in the colorectal cancer xenograft model. LOVO cells were subcutaneously injected into nude mice followed by control, low (20 mg/kg) or high (40 mg/kg) dose sAPRIL-BP injected intratumorally once the tumor was established. The mice were sacrificed at day 14, representative pictures of the mice and tumors are shown in Fig 6A. Treatment with sAPRIL-BP dose-dependently reduced the tumor volume (Fig 6A) and tumor weight (Fig 6B) in the mouse model. From day 8 of sAPRIL-BP injection, the tumor size of low and high dose groups was significantly *(p<*0.05) smaller than the control group (Fig 6C).

### Effect of sAPRIL-BP on cell proliferation and apoptosis in the xenograft tumor

**Fig 7:**
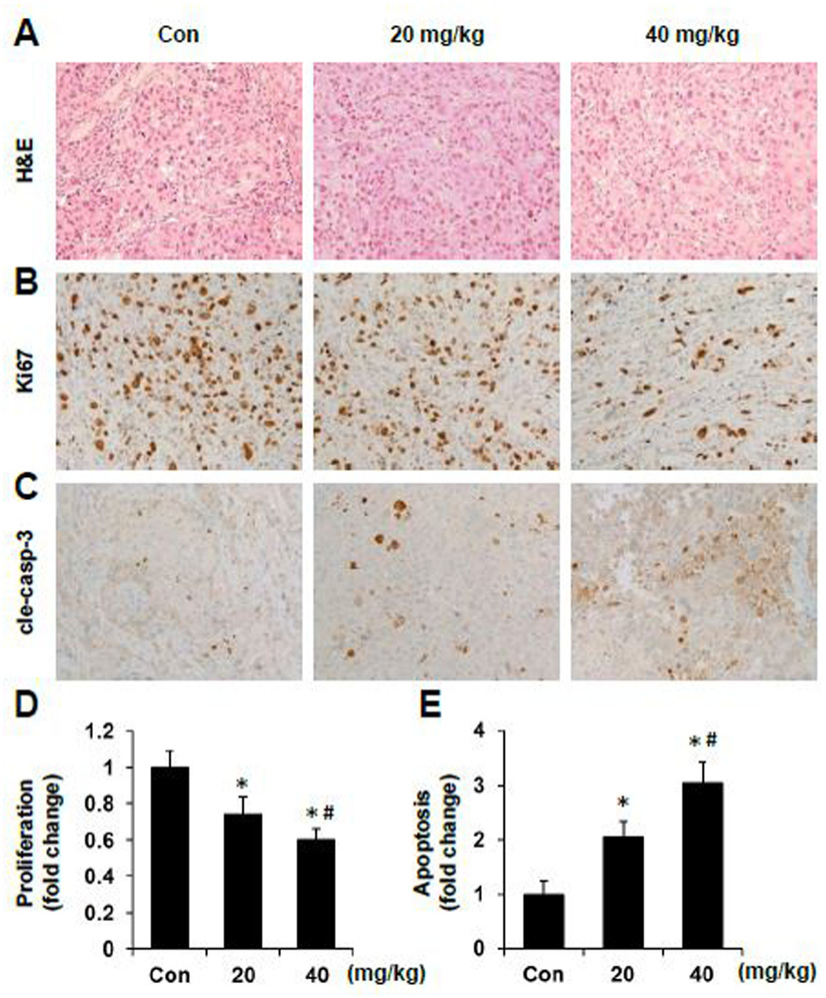
*In vivo* effect of sAPRIL-BP on proliferation and apoptosis in xenograft tumors. Paraffin embedded tumor tissues were used for morphological analysis with H&E staining (A), proliferation analysis with Ki67 staining (B), and apoptosis analysis with cleaved caspase-3 (cle-casp-3) staining (C). Representative pictures of the tumors from each group are shown (magnification 200×). The proliferation index (D) and apoptosis index (E) were calculated and normalized to the control (Con) group. *p <0.05 compared to control. #*p* <0.05 compared to 20 mg/kg group.

To further investigate the mechanism by which sAPRIL-BP inhibits xenograft tumor growth, we performed H&E staining (Fig 7A) on the tumor tissues and found that treatment with either low dose or high dose of sAPRIL-BP had no significant effects on tumor cell morphology and tumor mass architecture. A modest number of larger empty spaces were observed between tumor cells in the high dose group than the other two groups. Furthermore, immunohistochemical staining for the proliferation marker Ki67 on xenograft tumors (Fig 7B and 7D) showed sAPRIL-BP significantly inhibited cancer cell proliferation *in vivo* in a dose-dependent manner. In contrast, immunohistochemical staining for the apoptosis marker cleaved caspase-3 (Fig 7C and 7E) showed sAPRIL-BP treatment resulted in a significant and dose-dependent increase in apoptosis of tumor cells. These results indicated the sAPRIL-BP inhibit tumor growth through proliferation inhibition and apoptosis induction.

### Effect of sAPRIL-BP on the xenograft tumor metastasis

**Fig 8:**
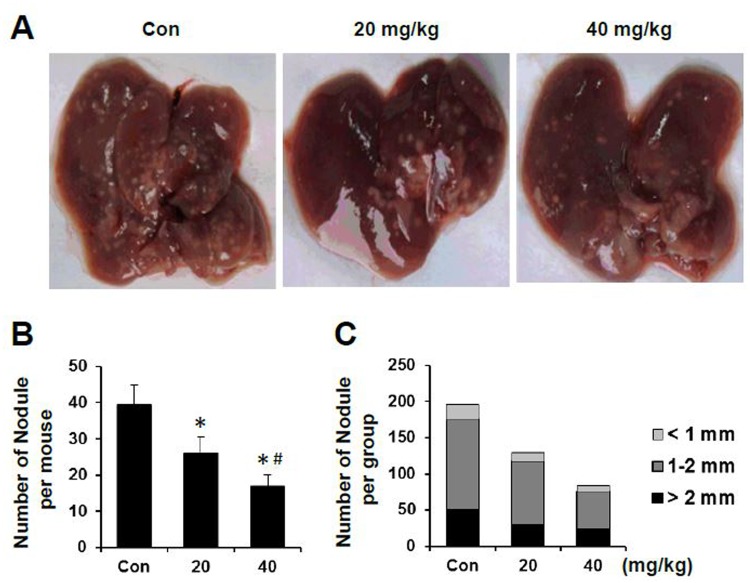
*In vivo* effect of sAPRIL-BP on liver metastasis. LOVO cells were injected into the spleens of nude mice to observe experimental liver metastasis. Three weeks after injection, the mice were divided into 3 groups (N = 5) and treated with PBS (control), low (20 mg/kg), or high (40 mg/kg) doses of sAPRIL-BP every other day. Mice were sacrificed after two weeks of treatment with sAPRIL-BP. (A) Representative pictures of the metastatic liver tumors from each group are shown. (B) Numbers of metastatic nodules per mouse were recorded. *P <0.05 compared to control. #*P* <0.05 compared to the low dose group. (C) Numbers of metastatic nodules with the indicated size were recorded. Total numbers of metastatic nodules: n = 197 (Con), n = 130 (20 mg/kg), and n = 84 (40 mg/kg).

To further investigate whether sAPRIL-BP treatment inhibit metastasis, we established a liver metastasis model by injecting LOVO cells into spleen. Representative pictures of the metastatic liver tumor were shown in Fig 8A. Treatment with sAPRIL-BP reduced the number of metastatic nodules in a dose-dependent manner (Fig 8B; *p*<0.05). The distribution of nodule sizes was similar in each group (Fig 8C). Taken together, these results suggested sAPRIL-BP treatment not only reduced tumor growth but also decreased liver metastasis in a mouse model of colorectal cancer.

## Discussion

In this study, we identified and characterized a sAPRIL-BP that could be a potential therapeutic for colorectal cancer. *In vitro* studies demonstrated both anti-proliferative and pro-apoptotic effects of sAPRIL-BP. Consistently, sAPRIL-BP reduced *in vivo* tumor growth by reducing intratumoral cell proliferation and increasing intratumoral cell apoptosis. Moreover, sAPRIL-BP significantly reduced liver metastasis of colorectal cancer cells in a mouse model. The effects of the sAPRIL-BP identified in this study will need to be tested further in clinical trials. Anti-cancer polypeptides have multiple advantages, such as easy synthesis and purification, low immunogenicity, and high penetration rate. This sAPRIL-BP could be a feasible strategy for treating APRIL^high^ cancers [34–38].

A phage display library contains a large number of diverse polypeptides expressed on the surface of phages [39]. This technique is widely used for screening drug targets, receptor agonist/antagonist, antigen epitope analysis, vaccine design, and analysis of protein structure [40–42]. We employed this method to develop sAPRIL specific binding peptides. Because one of the mechanisms underlying tumorigenesis is the disruption of the balance between cell proliferation and apoptosis, anti-cancer strategies that include inhibiting proliferation and/or inducing apoptosis in cancer cells are recommended [43, 44]. We hypothesized that the binding peptides, which compete with endogenous APRIL receptors to bind the soluble form of APRIL, would have therapeutic efficacy against APRIL^high^ cancers. Similar strategies targeting APRIL such as, using the soluble forms of decoy receptors to compete with ligand binding and silencing APRIL, have been shown to inhibit tumor growth in certain kinds of cancers. For instance, the soluble form of BCMA has been shown to inhibit the proliferative activity of APRIL *in vitro* and decrease tumor cell proliferation in nude mice [45]. Downregulation of APRIL by lentivirus-mediated RNAi effectively inhibits the growth of pancreatic cancer cells *in vitro* and *in vivo* [46]. Wang *et al*. [15, 26] also used siRNA to silence APRIL in a nude mouse colorectal cancer model and found that APRIL knockdown increased cancer cell apoptosis and reduced tumor growth and metastasis. Similarly, we have previously shown that lentivirus-mediated RNAi knockdown of APRIL inhibits the growth rate of LOVO cells and increases the number of cells in the G0/G1 phase and decreases the number in the G2/M phase [28]. Consistent with the previous work, this study showed that targeting APRIL using sAPRIL-BP has some efficacy against colorectal cancer lines.

Previous work [14, 21, 22, 24] suggested that the anti-apoptotic mechanisms of sAPRIL-BP might be attributable to blocking NF-κB signaling, and the regulation of anti-apoptotic proteins (Bcl-2, Bcl-xL, XIAP, ERK, and TGF-β) or pro-apoptotic proteins such as Bax. However, the detailed molecular mechanisms through which sAPRIL-BP increased apoptosis in LOVO cells remain to be further investigated. In addition, the sAPRIL-BP we identified showed specific binding affinity with sAPRIL and significant ability to competitively inhibit sAPRIL binding to APRIL receptors on LOVO cells. HSPG is the only APRIL receptor found on LOVO cells (data not shown). Therefore, whether sAPRIL-BP and HSPG share the same binding epitopes on sAPRIL, and the structural similarity between HSPG and sAPRIL-BP requires additional study.

APRIL- regulated cell proliferation has been implicated in many different cancers, but to date there are few published studies that demonstrate the molecular mechanism underlying APRIL-mediated cell cycle regulation. Wang *et al*. demonstrated a reduction in cyclin D1 and CDK4, which are critical regulators of the G1/S transition, when APRIL was knocked down in colorectal cancer cells. These findings suggested that cyclin D1 and CDK4 might be involved in APRIL-mediated regulation of cell proliferation [24, 25, 47]. Our results also demonstrated that sAPRIL-BP reduced the expression of CDK4 and cyclin D1 in a dose-dependent manner in the colorectal cancer LOVO cell line. However, it is likely that APRIL regulates the cell cycle through different mechanisms in cell type-dependent manner. For example, in multiple myeloma cells, APRIL promotes cell cycle progression in cyclin D2-positive cells by upregulating CDK4, CDK6, and phospho-retinoblastoma protein, but did not regulated cell-cycle proteins in cyclin D1-positive cells [23]. In gastric cancers, APRIL knockdown causes cell cycle arrest at the G2/M transition but not at the G0/G1 phase transition [48]. In hepatocarcinoma cells, an herbal medicine, *Blumea balsamifera*, has been shown to have anti-proliferative effects by reducing APRIL, which promotes cell cycle arrest in the G1 phase by decreasing the expression of cyclin-E and phosphorylation of retinoblastoma protein but not cyclin D1 and CDK4 [49]. Taken together, these results strongly suggest that APRIL has a common role regulating the cell cycle in cancer cells but that the underlying molecular mechanisms may vary depending on the type of cancer.

## Conclusions

This study successfully identified a specific peptide with high binding affinity to sAPRIL. *In vitro* and *in vivo* studies demonstrated that the sAPRIL-BP has significant anti-proliferative and pro-apoptotic effects against APRIL^hi^ colorectal cancer cells. The sAPRIL-BP might suppress proliferation by inhibiting cell cycle progression and upregulating cyclin D1 and CDK4. The results from this study suggest that targeting APRIL with sAPRIL-BP could be a novel therapeutic strategy for treating colorectal cancers with high levels of APRIL expression.
